# "For the Complexion" : A Rival to Pears

**Published:** 1890-04-26

**Authors:** 


### The Doctor's Pulpit.

### "FOR THE COMPLEXION:" A RIVAL TO PEARS.

Those young ladies who are fortunate enough to possess a good complexion value it highly, and those who are destitute of this peculiar mark of beauty covet it much. The desire for good looks is eminently commendable. If a woman were to tell the writer that she spent an hour a day in beautifying herself he would decidedly approve both her motive and her conduct, provided that she set about the attainment of her object in a rational way. Many persons consider it evidence of a vain mind to wish for good looks. But let us think about the thing scientifically. Here is a painter, painting little Lord Fauntleroy, and taking an infinity of pains to make his face, his hair, his arms, his hands, his legs, his feet, his attitude, his clothes, and everything about him an ideal of perfection. Does anyone blame the painter for this? Nay, rather would not everybody say that he was altogether unfit to paint the portrait of a child at all unless he did his best to make it a model of childish beauty? There is a mother, the wife of a village labourer, preparing her little girl for school. Is it not a positive pleasure to behold the clean and rosy face, the nicely brushed hair, tied though it may be with a piece of ribbon from which both colour and newness have long since departed; the spotless and neatly ironed pinafore, patched in half-a-dozen places; the well darned stockings and cobbled but not unpolished boots—is it not, we say, a pleasure to every wholesome mind to see this loving mother trying her best to realize God's idea, and to make her little one beautiful? Even the tailor, though only the ninth part of a man, will walk round and round, and view with critical eye the most unpromising of aldermanic figures, and will resolve, by toning up a little here and toning down a little there to make his customer's shape, if not that of an Apollo, at least that of a presentable City magnate.

Well then, if these people are doing but their plain and evident duty when they spend time and labour in trying to make others beautiful, by what law of reason or of religion is it forbidden to a woman, young or old, to labour to improve her own looks? It is forbidden by no law. None but the sour fanatic or the ugly cynic will so much as think of questioning the justice of what we say. By all means let women, and men, too, attach importance to their looks, and do all that knowledge can suggest and effort accomplish to produce the best possible results.

A good complexion is usually as plain an indication of good health as smoke from the chimney is of fire in the grate. The majority of town people have no complexions at all to speak of. This is due partly to the grime that adheres to the skin, but much more to the deficiency of oxygen in the air. Of all the conditions upon which a good complexion depends, oxygen is the chief. A woman can neither be well nor look well without an abundant supply of oxygen. Sitting rooms and bedrooms, however large they may be, never contain that proportion of pure oxygen which the complexion demands. The air of the streets is better than that of houses, but in London and other large towns even it cannot touch the face with the tints of beauty. May-dew is the thing. If a "peck of March dust be worth a king's ransom," a pint of May-dew is worth a queen's dowry. But the dew must be employed under right conditions. It will not do for the chamber-maid to bring it to the bedroom door in a jug when she brings up the hot water. May-dew is like certain facts of science, and can only be properly dealt with *in situ*, that is, in its own proper place and in its natural surroundings. As everybody knows, it need not be sought for in the streets of towns, nor even in the little strips of ground dignified by the name of "gardens" at the backs of the houses. Doubtless a fluid of some kind may be found even on the withered leaves and grass of large cities; but this is "town-dew," and has no more kinship with the genuine article than a "shop-un," as Middlewick called it, has with a fresh country egg. In order to get a May-dew that shall be properly and promptly efficacious it is necessary to look for it in the open fields, and at early morn. But here let a word of caution be said. The dew is not to be used "fasting." It may be sought for at seven o'clock in the morning or even at six, and there is no harm in walking one or two miles to find it. But remember, young lady, that it will do you no good whatever unless you take before you start one or two good thick slices of bread and butter and a cup of hot milk or of weak coffee, or tea, or cocoa. Perhaps the cocoa need not be weak, and probably it will be more sustaining than either coffee or tea. If the distance to the dew fields be great, a couple of fresh eggs, beaten up into a nice warm egg-flip, may be taken with the bread and butter instead of the coffee or tea. A plan like this religiously carried out for three months would almost put a complexion upon the ceiling of a London lodging house. Certainly no young lady, if she spends the remainder of the day reasonably well, can possibly find it to fail.

Nature, it is thus seen, can do much towards making a good complexion. What can art do? This is delicate ground; it is ice almost too thin for skating upon. Nevertheless we will make a bold venture. Whose art is to be relied upon for the production of a fresh and beautiful face? Your own! Nobody else's. Here is a great truth of science: The complexion of the mind has a powerful influence upon the complexion of the face. Nothing conduces more to health than a kind, obliging, unselfish, and cheerful disposition; and, by parity of reasoning, nothing conduces more to a beautiful complexion and good looks. But there is no art in this you will say. Is there not? It is one of the most skilful arts in the world to develop and maintain a kind, obliging, unselfish, and cheerful disposition. If you try it you will soon find out. Now this is eminently true and eminently scientific; much more scientific than lotions, and skin washes, and powders, and puffs, and all that kind of cheating knavery and unscientific villainy. Nature is a wise, gentle, and perfect teacher of science. Obey her; learn of her; love her with an ardent enthusiasm. She is to be admired in the dewy fields at early morning, and in the best dispositions of your own heart during the day. If she were but always admired and obeyed, ugly people and unhealthy people would soon be almost unknown.

